# Hemolytic anemia induced by malpositioned covered stent following acute type A aortic dissection repair

**DOI:** 10.1093/ehjcr/ytaf661

**Published:** 2025-12-22

**Authors:** Shanliang Chen, Xiaozhou Zheng, Huimin Cui

**Affiliations:** Department of Cardiovascular Surgery, The First Affiliated Hospital of Shandong First Medical University (Shandong Provincial Qianfoshan Hospital), No. 16766, Jingshi Road, Jinan 250014, China; Department of Cardiovascular Surgery, The First Affiliated Hospital of Shandong First Medical University (Shandong Provincial Qianfoshan Hospital), No. 16766, Jingshi Road, Jinan 250014, China; Department of Cardiovascular Surgery, The First Affiliated Hospital of Shandong First Medical University (Shandong Provincial Qianfoshan Hospital), No. 16766, Jingshi Road, Jinan 250014, China

## Case description

A 57-year-old male with a history of Bentall, Sun's procedure and left atrial appendage clip occlusion performed 3 months prior was admitted for haemolytic anaemia. Aortic computed tomography angiography (CTA) and transthoracic echocardiography (TTE) revealed coronary anastomotic leaks via the aortic wrap to the right atrium: one left-sided (3.3 mm) and two right-sided (2.9 and 1.2 mm) (*Panels A* and *B*).

**Figure:**
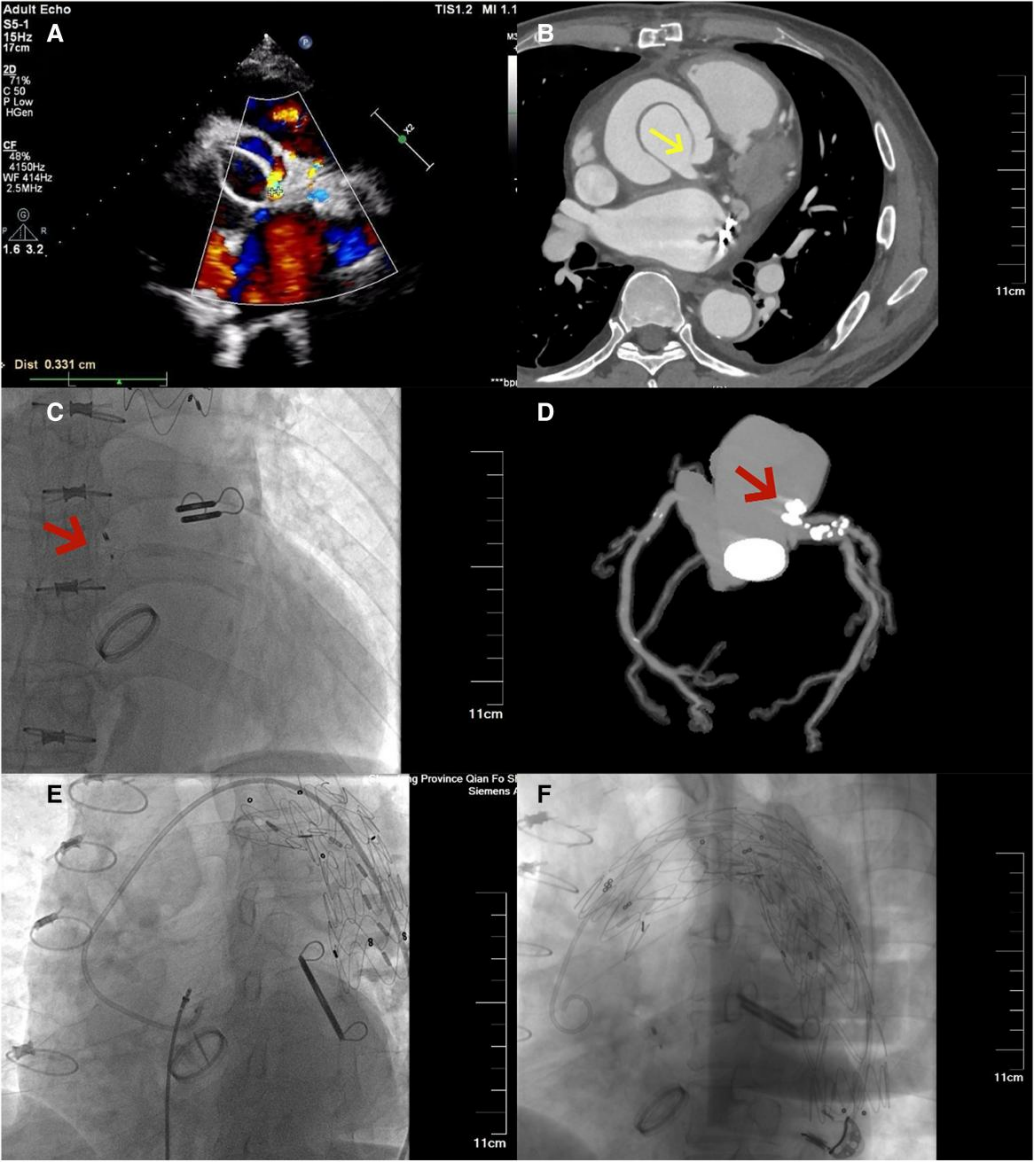


Digital subtraction angiography (DSA)-guided leak occlusion was performed. A 5-mm ventricular septal defect occlude (Lifetech Scientific, Inc. Shenzhen, China) successfully sealed the primary left leak (*Panels C* and *D*); however, intra-operative DSA fluoroscopy demonstrated a retrograde placed stent silhouette at the distal aortic arch, which swung continuously in the distal aortic arch throughout the cardiac cycle (*Panel E*), causing mechanical destruction of blood, suggesting a causal relationship with haemolytic anaemia. Further history review revealed that the initial frozen elephant trunk (FET) procedure was performed using a 30-30-080 covered stent (Weiqiang Medical Tech, Co., Ltd, Hangzhou, China). However, it was retrograde placed with its morphological distal end anchored within a 28-mm Four-Branched artificial vascular graft at the distal aortic arch. One month later, thoracic endovascular aortic repair (TEVAR) was performed to deploy a covered stent (Medtroni, 30-30-150) to secure the swinging stent to prevent any further movement (*Panel F*). A compliant balloon was then advanced to the malpositioned stent segment and inflated to remodel the stent-graft. At 1-month follow-up, haemoglobin levels trended from 56 g/L (second admission) to 50 g/L (third admission), rising to 102 g/L recently.

Haemolytic anaemia is a catastrophic complication following acute type A aortic dissection repair. Severe anaemia may necessitate transfusion, and its management remains clinically challenging.

## Data Availability

The data underlying this article will be shared upon reasonable request to the corresponding author.

